# Quality of life in patients with a permanent stoma after rectal cancer surgery

**DOI:** 10.1007/s11136-016-1367-6

**Published:** 2016-07-21

**Authors:** Pia Näsvall, Ursula Dahlstrand, Thyra Löwenmark, Jörgen Rutegård, Ulf Gunnarsson, Karin Strigård

**Affiliations:** 1 Department of Surgery, Sunderby Hospital/Umeå University, 97180 Luleå, Sweden; 2 Department of Surgical and Perioperative Sciences, Umeå University, Umeå, Sweden; 3 Department of Clinical Sciences, Intervention and Technology, CLINITEC, Karolinska Institute, Stockholm, Sweden

**Keywords:** HRQoL, Stoma, Parastomal, Hernia, Rectal cancer

## Abstract

**Aim:**

Health-related quality of life (HRQoL) assessment is important in understanding the patient’s perspective and for decision-making in health care. HRQoL is often impaired in patients with stoma. The aim was to evaluate HRQoL in rectal cancer patients with permanent stoma compared to patients without stoma.

**Methods:**

711 patients operated for rectal cancer with abdomino-perineal resection or Hartman’s procedure and a control group (*n* = 275) operated with anterior resection were eligible. Four QoL questionnaires were sent by mail. Comparisons of mean values between groups were made by Student´s independent *t* test. Comparison was made to a Swedish background population.

**Results:**

336 patients with a stoma and 117 without stoma replied (453/986; 46 %). A bulging or a hernia around the stoma was present in 31.5 %. Operation due to parastomal hernia had been performed in 11.7 % in the stoma group. Mental health (*p* = 0.007), body image (*p* < 0.001), and physical (*p* = 0.016) and emotional function (*p* = 0.003) were inferior in patients with stoma. Fatigue (*p* = 0.019) and loss of appetite (*p* = 0.027) were also more prominent in the stoma group. Sexual function was impaired in the non-stoma group (*p* = 0.034). However in the stoma group, patients with a bulge/hernia had more sexual problems (*p* = 0.004). Pain was associated with bulge/hernia (*p* < 0.001) and fear for leakage decreased QoL (*p* < 0.001). HRQoL was impaired compared to the Swedish background population.

**Conclusion:**

Overall HRQoL in patients operated for rectal cancer with permanent stoma was inferior compared to patients without stoma. In the stoma group, a bulge or a hernia around the stoma further impaired HRQoL.

## Introduction

Health-related quality of life (HRQoL) estimates are necessary to understand the patient’s perspective as well as for decision-making and planning of health care. Historically surgical outcomes, such as complications, tumor response, survival and relapse, have been the most important end points. Surgical outcomes have improved over time in several aspects. The recurrence rate of rectal cancer has decreased to <10 % [1], compared to earlier figures of up to 30 % [2], and survival after rectal cancer has also improved. As more patients survive, with reduced risk for recurrent disease, the HRQoL has become increasingly important. Moreover, the legislation of patients´ involvement in the decision of treatment options further stresses the importance of tools for HRQoL.

The HRQoL estimates represent a crucial feedback system to the physician and are helpful when implementing new as well as when evaluating already established techniques [3].

Surveys can be divided into general health and disease-specific questionnaires, the former covering aspects concerning a broad spectrum of health and daily life. Country-specific estimates from the healthy population, facilitating interpretation of patient estimates, are available for most general validated questionnaires. Unexpected side effects to treatment can be found using these general questionnaires [4]. Disease-specific questionnaires concentrate on health-related issues associated with the given disease.

The incidence of parastomal hernia is still not established; however, the frequencies range from a few percent up to 78 % [5, 6]. Parastomal hernia can give the patient a bulge around the stoma, but on the other hand some parastomal hernias are subclinical and some bulges do not correspond to a hernia. Patients often experience a bulge as being inconvenient, sometimes due to difficulties with stoma appliances. If the bulge represents a hernia, it might be possible to offer surgical treatment. HRQoL has been shown to be impaired in patients with a stoma, especially with one that easily leaks or with complications like parastomal hernia [7, 8]. On the contrary, there are studies challenging the assumption that patients with a stoma perceive inferior HRQoL [9]. As cancer and cancer-associated treatment also can affect HRQoL [10], this must be taken into account.

In Sweden, it is mandatory by law to register all patients operated for rectal cancer in the National Cancer Registry (NCR). Patients are reported by both the clinician and the pathologist. Registrations in the national quality register started in 1995 [1], the Swedish Rectal Cancer Register (SRCR), and are checked for completeness against the NCR. Although patients have the possibility to decline participation in the SRCR, the completeness was 99 % in 2013. Approximately, 2000 new rectal cancers are diagnosed annually in Sweden and almost 90 % of these are operated. During the period 1996–2004, approximately one-third of the surgically treated patients were operated with a permanent stoma [abdomino-perineal resection (APR) or Hartman’s procedure (HA)], whereas two-thirds were operated with an anterior resection (AR). In addition to this, a large proportion of patients are operated with low anterior resection and temporary loop ileostomy, and approximately 20 % of this group never have their stoma reversed.

The aim of this study was to evaluate HRQoL in rectal cancer patients with permanent stoma. The hypothesis was that stoma-related complaints and complications decrease HRQoL.

## Methods

### Study design

A cross-sectional study of HRQoL among Swedish patients operated for rectal cancer with or without a permanent stoma was performed.

### HRQoL questionnaires

Four HRQoL questionnaires were used: EORTC QLQ-C30, EORTC QLQ-CR38, SF-36 and Colostomy Questionnaire (CQ).

Two of these questionnaires were developed by the European Organisation for Research and Treatment of Cancer (EORTC) [11]. EORTC QLQ-C30 is designed to assess the quality of life of cancer patients overall, whereas EORTC QLQ-CR38 is a disease-specific module for colorectal cancer. Both these instruments are validated and available in Swedish [12].

The EORTC QLQ-C30 module consists of 30 items and includes scales measuring global health, functioning scales (physical, emotional, cognitive and social) and single-item scales (dyspnea, insomnia, loss of appetite, bowel function and financial impact). EORTC QLQ-CR38 concentrates on cancer-specific questions and is constituted of 38 items. Questions are grouped in four functioning scales (body image, future perspectives, sexual functioning and sexual enjoyment) and eight symptom scales (urinary, gastrointestinal, defecation, sexual, chemotherapy side effects, weight loss and stoma related).

SF-36 health survey by Medical Outcome Trust (MOT) is a validated [13] HRQoL questionnaire available in Swedish [14]. It constitutes 36 items concerning physical functioning and role, bodily pain, general health, vitality, social functioning, emotional role and mental health.

The CQ is an [15] HRQoL instrument for stoma patients comprising 30 items. The topics cover if and how the patient is affected by the stoma with regard to daily life, physical activities, profession, sexuality, pain, bulging around the stoma, urinary continence and limitations in daily life. One question in CQ was “Do you have a bulge or a hernia around your stoma?”. Results in CQ correspond to the clinical presentation, but there is still a divergence in the interpretation of clinical assessments implicating that answers represent the patients’ perception [15]. Strict validation cannot be done due to uncertainty regarding interpretation in clinical and computed tomography assessments.

EORTC QLQ-C30 and CR38 were interpreted according to the scoring manual [16]. The QLQ-C30 and CR 38 are composed of multi-item and single-item questions, all with four- to seven-category answer options. Both questionnaires are re-scaled from 0 to 100. This means that a high score for a functional scale corresponds to a high or healthy level of functioning and a high global health status corresponds to a high HRQoL. In contrast, a high score in a symptom or single-item scale represents a high level of symptoms. EORTC QLQ-C30 data from 2000 in an age and gender adjusted healthy Swedish population was used for comparison [17].

SF-36 license including software for scaling and scoring of data was obtained. The questionnaire is composed of eight domains of items and two component summaries. Scores are weighted and transformed into a scale from 0, representing worst possible health or severe disability, to 100 representing the best possible health or no disability. Data from 1994, representing a normal Swedish background population for the age cohort 70–74 years, was used for comparison [14].

CQ consists of 23 items with five- to six-category answer options, 5 items with a yes or no answer and 2 items describing the operation technique and symptoms related to an intact anus. The Mean values for the 23 items are calculated.

### Patients

Patients operated for rectal cancer during 1996–2004, identified in SRCR, who were alive in 2008 were eligible. Inclusion criteria were: patients operated with APR and HA in the Uppsala/Örebro, Stockholm/Gotland and Northern Regions with the exception of those operated in Karolinska-Solna Hospital and Sundsvall Hospital. The reason for excluding patients operated in the later hospitals was the routine use of a prophylactic mesh when creating a permanent stoma. 711 patients (54.9 % male, 45.1 % female) met the inclusion criteria and received the four HRQoL questionnaires.

A control cohort of patients without a permanent stoma was collected from the Northern Region by including 275 patients (55.6 % male, 44.4 % female) operated with AR. 88 patients had a temporary loop ileostomy and 31 of these were reversed before 2008; thus, 57/275 (20.1 %) still had a loop ileostomy at the time of the questionnaire survey. These patients were considered as having permanent stomas and were thus included in the stoma group and not as control cases. Patient characteristics were retrieved from the SRCR.

Addresses were obtained by the PAR AB Company, the Swedish mail company. The questionnaires were sent by mail by the Mailit Company with attached response envelopes to a total cohort of 986 patients in the year 2009. A reminder was sent to non-responders after 6 months.

The study protocol adheres to the Helsinki Declaration and was approved by the Ethics Committee at Uppsala University, Sweden 2005:287.

### Statistics

Data were collected in an Access^®^ database and the IBM SPSS Statistics 22 software package was used for statistical analysis. Comparisons of mean values between groups were made using the independent Student’s *t* test. For all HRQoL forms, a *p* value below 0.05 was considered significant. For multivariate analysis, “univariate analysis of variance” was used and adjustment for age and gender made.

## Results

**Fig. 1:**
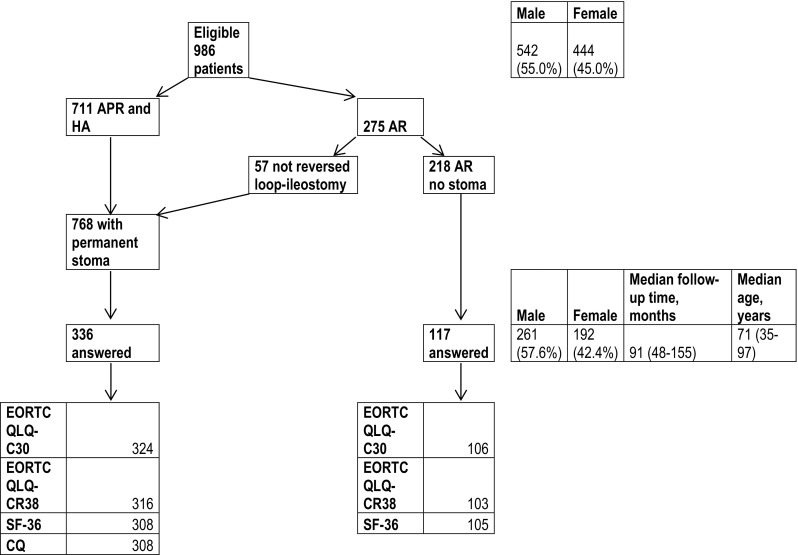
Diagram showing patients receiving HRQoL forms and distribution of answers. Gender, age and follow-up time in eligible patients and patients replying to the forms are shown on the right

Answers were obtained from 453 participants, representing a response rate of 46 %. In total, 336/453 (74.2 %) of the patients had a permanent stoma: 281 had a permanent colostomy and 55 had a non-reversed loop ileostomy and were thus regarded as permanent stomas The median age in this group was 71 years (35–97). 117 (25.8 %) of the patients had a colorectal or coloanal anastomosis with a median age of 71 years (37–89). A higher proportion of male patients answered the questionnaires: 261 males (57.6 %) and 192 (42.4 %) females, which reflects the original male/female (55/45 %) rate. Median follow-up time after rectal cancer surgery was 91 months (48–155), 90 months (48–155) in the stoma group and 93 months (49–155) in the non-stoma group (Fig. 1). 430 patients answered the EORTC QLQ-C30 questionnaire (324 with a stoma and 106 without permanent stoma) and 419 answered the EORTC QLQ-CR38 questionnaire (316 with a stoma and 103 without permanent stoma). 308 patients with a permanent stoma answered the CQ questionnaire. Of the 413 patients answering the SF-36 form, 308 had a permanent stoma. The distribution of answers is shown in Fig. 1. In the group not responding to the HRQoL (533 patients), the median age was 74 years (35–87) and median follow-up time was 95 months (50–158).

**Fig. 2:**
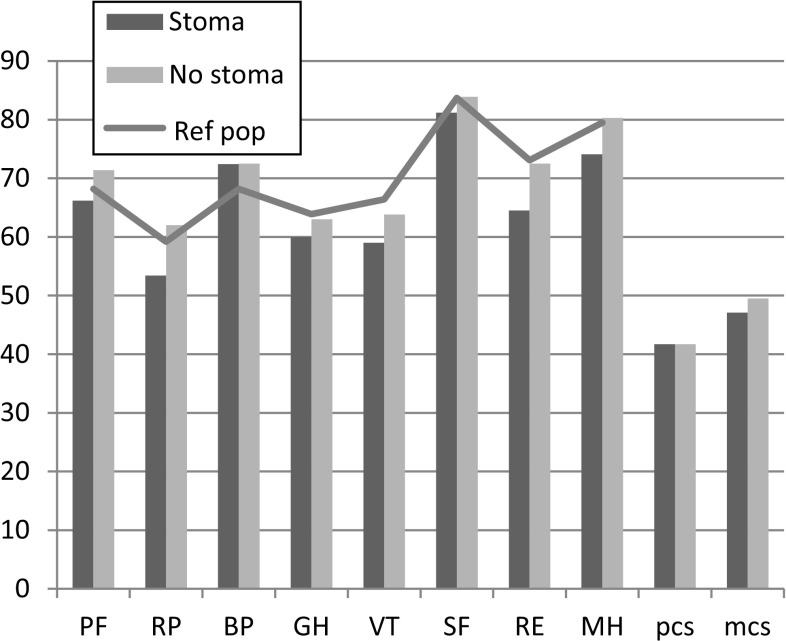
SF-36 mean values in the group with permanent stoma and without permanent stoma. The *line* represents the healthy age-matched Swedish population (Ref pop). *PF* physical functioning, *RP* physical role functioning, *BP* bodily pain, *GH* general health, *VT* vitality, *SF* social functioning, *RE* emotional role functioning, *MH* mental health, *pcs* physical component summary, *mcs* mental component summary. High mean value represents good health or no disability

Comparison of mean values between the stoma and non-stoma group for SF-36 scorings showed higher mental health (MH) ratings (*p* = 0.007) in the group without stoma. Mental component summary (mcs) ratings had a tendency to be better in the group without stoma. Stoma group patients had lower ratings compared to the Swedish “normal population” except in bodily pain (BP), where the Swedish “normal population” seemed to have more pain. The non-stoma group scored similar to the Swedish “normal population” in most domains. However, vitality (VT) and general health (GH) seemed, but not significantly, to be better in the Swedish “normal population” compared to both the stoma and non-stoma groups (Fig. 2; Table 1). Multivariate analysis adjusting for age and gender revealed higher rating for mental health (MH, *p* = 0.059) in the group without stoma (Table 1).

**Table 1:** SF-36 comparison of mean values and *t* tests in the groups with and without stoma

	Mean value	*t* test sign	*n*	Gender, age adjusted
PF physical functioning				
Stoma	66.2	0.097	317	0.447
No stoma	71.4		104	
RP physical role functioning				
Stoma	53.4	0.089	321	0.653
No stoma	62.0		101	
BP bodily pain				
Stoma	72.4	0.962	317	0.412
No stoma	72.6		104	
GH general health				
Stoma	60.0	0.265	312	0.916
No stoma	63.0		100	
VT vitality				
Stoma	59.0	0.080	312	0.649
No stoma	63.8		102	
SF social functioning				
Stoma	81.2	0.346	315	0.774
No stoma	83.9		104	
RE emotional role functioning				
Stoma	64.5	0.103	307	0.498
No stoma	72.5		100	
MH mental health				
Stoma	74.1	0.007	314	0.059
No stoma	80.3		102	
Pcs physical component summary				
Stoma	41.7	0.260	299	0.547
No stoma	43.2		95	
Mcs mental component summary				
Stoma	47.1	0.069	299	0.739
No stoma	49.5		95	

Results for multivariate analyses adjusted for age and gender are presented at the far right. *p* values are presented for *t* test and gender, age adjusted

**Fig. 3:**
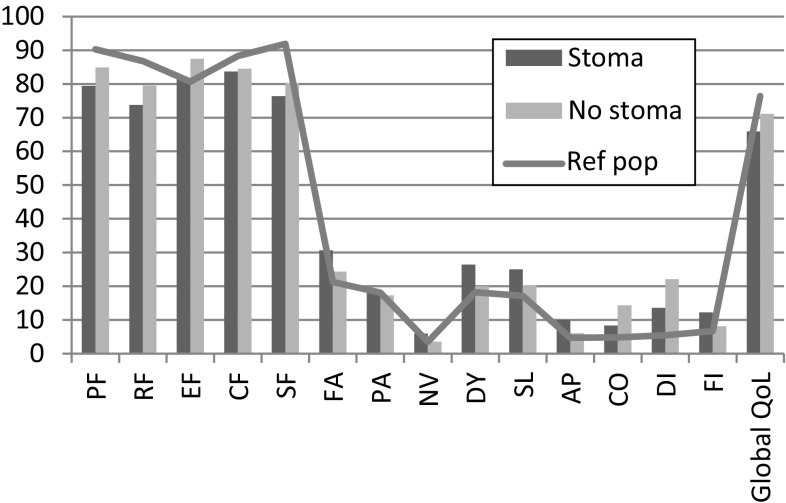
EORTC QLQ-C30 mean values in the group with permanent stoma and the group without permanent stoma. The *line* represents the healthy Swedish population, adjusted for age and gender (Ref pop). Functional scales: *PF* physical function, *RF* role function, *EF* emotional function, *CF* cognitive function and *SF* social function. Symptom scales: *FA* fatigue, *PA* pain, *NV* nausea and vomiting. Single-item scales: *DY* dyspnea, *SL* insomnia, *AP* loss of appetite, *CO* constipation and *FI* financial impact. *Global QoL* global health status. High mean value in *functional scales and global QoL* represents high or healthy level of functioning and QoL. High mean value in symptom and *single-item scales* represents a high level of symptoms

**Fig. 4:**
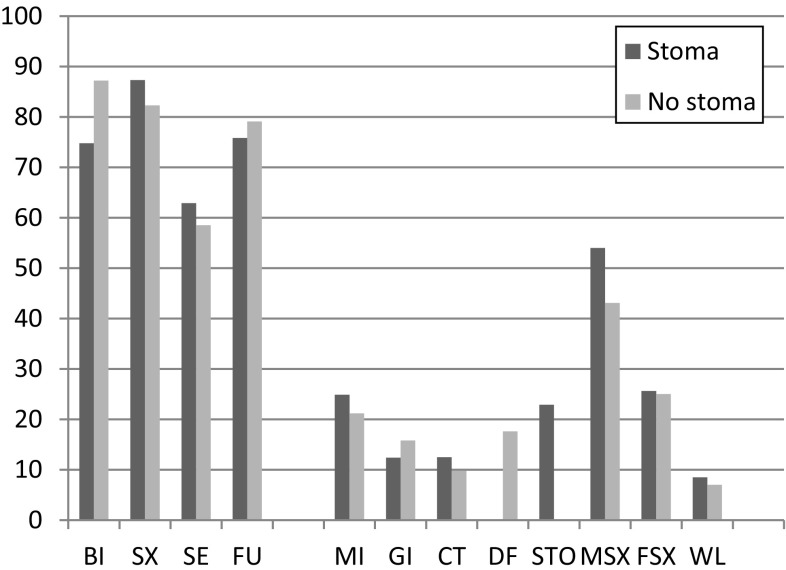
EORTC QLQ-CR38 mean values in the groups with and without permanent stoma. Function scales: *BI* body image, *SX* sexual functioning, *SE* sexual enjoyment, *FU* future perspective. Symptom scales: *MI* micturition problems, *GI* gastrointestinal symptoms, *DF* defecation problems (only for patients without stoma and intact sphincter), *STO* stoma-related problems (only for patients with stoma), *MSX* male sexual problems, *FSX* female sexual problems and *WL* weight loss. High mean value in *function scales* represents high or healthy level of functioning. High mean value in *symptom scales* represents high level of symptoms

EORTC QLQ-C30 showed higher scores for physical (PF, *p* = 0.016) and emotional (EF, *p* = 0.003) function for patients operated without stoma. Stoma patients scored higher for fatigue (FA, *p* = 0.019), dyspnea (DY, *p* = 0.038) and loss of appetite (AP, *p* = 0.027). Diarrhea (DI, *p* = 0.012) and constipation (CO, *p* = 0.017) were more pronounced in the group without stoma. A higher, but not significant, degree of financial impact (FI, *p* = 0.081) and inferior global QoL (*p* = 0.052) was shown for patients with permanent stoma. Both the stoma and the non-stoma group (Fig. 3; Table 2) scores showed generally impaired health compared to the “normal” Swedish population in the year 2000 [17]. In the multivariate analysis (Table 2), constipation (CO, *p* = 0.017) and diarrhea (DI, *p* < 0.001) showed similar results. Financial impact (FI, *p* = 0.031) showed significant impact when adjusting for age and gender. The EORTC QLQ-CR38 showed higher ratings for body image (BI, *p* < 0.001, *p* < 0.001 gender and age adjusted), but sexual functioning (SX, *p* = 0.034) was worse in patients operated without stoma (Fig. 4; Table 3).

**Table 2:** EORTC QLQ-C30 comparison of mean values and *t* tests in the groups with and without permanent stoma

	Mean value	*t* test sign	*n*	Gender, age adjusted
*Functional scales*				
PF physical function				
Stoma	79.4	0.016	323	0.447
No stoma	84.9		106	
RF role function				
Stoma	73.8	0.092	322	0.592
No stoma	79.5		104	
CF cognitive function				
Stoma	83.7	0.720	322	0.665
No stoma	84.5		99	
EF emotional function				
Stoma	81.4	0.003	322	0.068
No stoma	87.5		99	
SF social function				
Stoma	76.4	0.207	321	0.774
No stoma	80.3		99	
*Symptom scales*				
FA fatigue				
Stoma	30.6	0.019	323	0.566
No stoma	24.3		106	
PA pain				
Stoma	18.7	0.624	322	0.194
No stoma	17.3		106	
NV nausea and vomiting				
Stoma	5.9	0.104	323	0.410
No stoma	3.4		106	
*Single item*				
DY dyspnoea				
Stoma	26.4	0.038	317	0.829
No stoma	20.0		105	
SL insomnia				
Stoma	25.0	0.154	323	0.346
No stoma	20.3		105	
AP loss of appetite				
Stoma	10.4	0.027	322	0.254
No stoma	6.0		106	
CO constipation				
Stoma	8.3	0.017	318	0.017
No stoma	14.3		105	
DI diarrhea				
Stoma	13.6	0.012	319	<0.001
No stoma	22.1		98	
FI financial impact				
Stoma	12.3	0.081	321	0.031
No stoma	8.1		99	
*Global health status*				
QL2 Global QoL				
Stoma	65.9	0.052	322	0.782
No stoma	71.1		98	

Results for multivariate analyses adjusted for age and gender are presented at the far right. *p* values are presented for *t* test and gender, age adjusted

**Table 3:** EORTC QLQ-CR38 comparison of mean values and *t* tests in the groups with and without permanent stoma

	Mean value	*t* test sign	*n*	Gender, age adjusted
*Function*				
BI body image				
Stoma	74.8	<0.001	314	<0.001
No stoma	87.2		103	
SE sexual functioning				
Stoma	87.3	0.034	298	0.207
No stoma	82.3		92	
SE sexual enjoyment				
Stoma	62.9	0.357	85	0.347
No stoma	58.5		41	
FU future perspective				
Stoma	75.8	0.195	307	0.340
No stoma	79.1		103	
*Symptoms*				
MI micturition problems				
Stoma	24.9	0.173	315	0.345
No stoma	21.2		102	
MSX male sexual problems				
Stoma	54.0	0.016	160	0.383
No stoma	43.1		38	
FSX female sexual problems				
Stoma	25.6	0.950	20	0.668
No stoma	25.0		15	
WL weight loss				
Stoma	8.5	0.445	314	0.748
No stoma	7.0		103	

Results for multivariate analyses adjusted for age and gender are presented at the far right. *p* values are presented for *t* test and gender, age adjusted

**Fig. 5:**
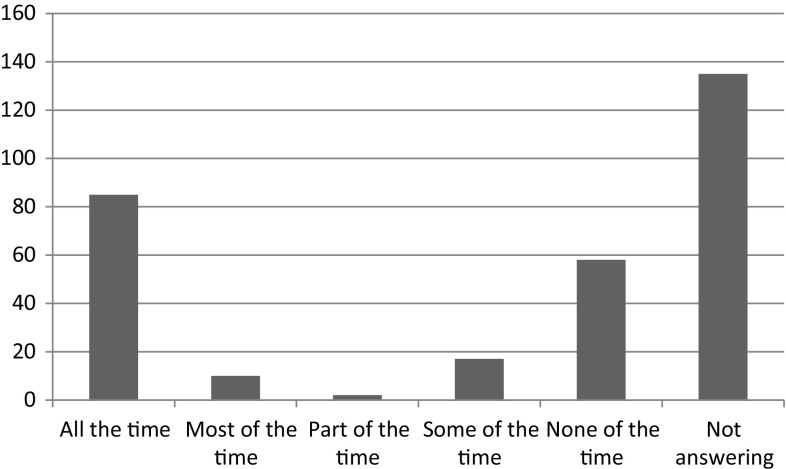
Patients with permanent stoma answering the question in CQ questionnaire: Do you have a bulge or a hernia around your stoma?

When patients with a permanent stoma operated with APR or HA answered the question in CQ; “Do you have a bulge or a hernia around your stoma?”, 97 patients stated a bulge or a hernia “part of the time” to “all of the time”, whereas 76 patients stated that they did not experience hernia or a bulge. 135 patients did not answer the question (Fig. 5). The prevalence of a bulge or a parastomal hernia in this group was 31.5 % (97/308). According to the answers in CQ, 36/308 (11.7 %) patients were operated due to parastomal hernia. Though operated due to parastomal hernia, half of this group stated having a bulge/hernia around the stoma. The hernia was repaired with mesh in 17 cases, local tissue repair in 7 cases and stoma relocation in 8 cases. In four cases, the method of repair was not given. Furthermore, 64/308 (20.8 %) patients needed to seek acute health care due to stoma complaints not related to bulge or no bulge.

Mean values were compared between the group with a permanent stoma and with or without bulge/hernia around the stoma. Physical function (PF, *p* = 0.038) in EORTC QLQ- C30 and physical role functioning (RP, *p* = 0.033) in SF-36 were better in the group without bulge/hernia. Sexual enjoyment (SE) and sexual functioning (SX) tended to be inferior in the group with a bulge/hernia according to scorings from EORTC QLQ-CR38 (Table 4).

**Table 4:** Patients with permanent stoma with or without a bulge or a hernia around the stoma

Bulge/hernia	Mean value	*t* test	*n*
*C30*			
PF physical role functioning			
Yes	73.2	0.038	97
No	80.1		75
*CR38*			
SX sexual functioning			
Yes	84.1	0.051	88
No	90.1		70
SE sexual enjoyment			
Yes	56.2	0.061	19
No	71.1		24
*SF-36*			
RP physical role functioning			
Yes	41.8	0.033	95
No	56.6		72

There were significant or considerable mean value differences in the three QoL questionnaires. All other comparisons of mean values were not significant. *p* values are presented for *t* test

A hernia or a bulge around the stoma had significantly negative effect on sex life (*p* = 0.004) as well as psychological well-being (*p* = 0.002) according to CQ answers. Pain was significantly more associated with a bulge or hernia (*p* < 0.001) with negative impact on QoL. On the contrary, physical activity was negatively affected (*p* = 0.018) in those without a bulge/hernia (Table 5). When adjusting for age and gender in multivariate analysis, these findings remained significant (Table 5). The functionality of the stoma was significantly (*p* < 0.001) impaired by fear of leakage (Table 6).

**Table 5:** Questions in the CQ form answered by patients with permanent stoma with or without a bulge/herina around the stoma

Bulge/hernia	Mean value	*t* test sign	*n*	Gender, age adjusted
Health today				
Yes	2.23	0.994	78	0.506
No	2.23		35	
Functionality of the stoma				
Yes	1.92	0.699	78	0.510
No	1.86		35	
Frequency emptying the stoma				
Yes	2.90	0.538	78	0.466
No	2.77		35	
Does the stoma affect your daily life?				
Yes	3.09	0.053	97	0.046
No	3.57		75	
Did the stoma change your physical activities?				
Yes	1.64	0.018	97	0.012
No	0.93		75	
Did the stoma affect your psychological well-being?				
Yes	2.92	0.002	97	0.002
No	3.77		75	
Do you have pain connected to your stoma?				
Yes	2.22	<0.001	97	<0.001
No	3.44		75	
Do you have sexual problems after the stoma operation?				
Yes	1.28	0.004	97	0.006
No	2.07		75	

Results for multivariate analyses adjusted for age and gender are presented at the far right. *p* values are presented for *t* test and gender, age adjusted

**Table 6:** Questions in the CQ form answered by patients with permanent stoma with or without concern about leakage from the stoma

Concern for leakage	Mean value	*t* test sign	*n*
Health today			
Yes	2.38	0.941	80
No	2.39		146
Functionality of the stoma			
Yes	2.08	<0.001	80
No	1.59		146
Frequency emptying the stoma			
Yes	3.09	0.083	80
No	2.84		146
Does the stoma affect your daily life?			
Yes	2.87	0.559	101
No	3.01		185
Did the stoma change your physical activities?			
Yes	1.50	0.208	101
No	1.19		185
Did the stoma affect your psychological well-being?			
Yes	2.66	0.799	101
No	2.73		185
Do you have any pain connected to your stoma?			
Yes	2.06	0.546	101
No	1.89		185
Do you have sexual problems after the stoma operation?			
Yes	1.27	0.115	101
No	1.61		185

*p* values are presented for *t* test

No difference between genders was found correlated to the experience of a bulge or hernia around the stoma.

## Discussion

Overall HRQoL in patients operated for rectal cancer with permanent stoma was inferior compared to patients without permanent stoma. In the stoma group, a bulge or a hernia around the stoma had additional negative impact on HRQoL. There was impaired HRQoL in a lot of aspects compared to the “normal population” in Sweden, especially in the stoma group according to SF-36 and EORTC QLQ-C30.

A bulge can be difficult to distinguish from a parastomal hernia [15] and all hernias probably do not need surgical intervention. In this study, almost 12 % of stoma patients had been re-operated due to parastomal hernia. The prevalence of bulge/hernia in this cohort was at least 31.5 %. The true rate might be even higher as the question was answered by less than two-thirds of the stoma patients. This is further supported by the fact that half of the patients operated due to parastomal hernia reported having a bulge/hernia. However, compared to earlier studies [5, 6], these frequencies appear reasonable. Stoma-related complaints led to acute medical care for nearly 21 % of the stoma patients. To our knowledge, this is new information and the proportion patients needing acute medical care must be regarded as high.

The response rate is a weakness in this study, which makes it more difficult to interpret. On the other hand, all patients operated for rectal cancer in the defined catchment area from 1996 till 2004 who were still alive in 2008 were eligible. Studies often exclude patients with metastatic or recurrent disease, psychiatric conditions or have a selection bias by judgment from other physicians whether or not the patient is suitable for contact [18]. No such exclusions were made in this present study. One reason for a fairly low response rate might have been the amount of questions included in the four questionnaires. Another reason can be the fact that the operation was several years ago, making the patient feel that it was not important to participate.

EORTC QLQ-30 and SF-36 are both general health questionnaires and similar in their content. Earlier studies have shown good correlation between these two questionnaires among breast and colon cancer patients [18], proposing them to be comparable tools. The present study of rectal cancer patients also revealed a good correlation between EORTC QLQ-C30 and SF-36 responses. Thus, both formularies are comparable tools for measurement of HRQoL in rectal cancer as well. In future rectal cancer studies, the usage of one of these questionnaires will give sufficient information about general HRQoL. According to a recent review, EORTC questionnaires were scored to be the best HRQoL tools for colorectal cancer patients, indicating their preference [19]. Comparison with back-ground population has some limitations regarding the time lag in SF36 as the normative data are from the year 1994.

Scoring of body image was impaired by having a stoma, and a parastomal hernia or a bulging around the stoma increased this negative impact. Physical role functioning was significantly impaired as measured both by EORTC QLQ-C30 and SF-36. A possible explanation could be difficulties with stoma dressings or influence on physical activities due to the bulge or hernia, as shown in earlier studies [7]. According to the answers in CQ, the stoma affected physical activities to a significantly higher degree when the patient had a bulge or a hernia around the stoma. Fear of leakage clearly impaired the functionality of the stoma.

The stoma group experienced more fatigue and loss of appetite. Fatigue has been reported as a stress factor influencing daily life in patients with colostomy [20]. Concerns regarding the stoma and changing of stoma dressing might be one cause of impaired appetite. It may also be speculated that the stoma is a daily reminder of the earlier cancer. On the other hand, constipation and diarrhea were more pronounced among patients without a stoma. Diarrhea, fecal incontinence and emptying difficulties are symptoms related to a low anastomosis, often referred to as low anterior resection syndrome (LARS) [21].

There were no significant differences between the stoma and the non-stoma group regarding pain or bodily pain. However, a bulge or a hernia around the stoma gave significantly more pain. This might indicate a need for treatment of the bulge/hernia.

Earlier studies point out sexual problems in patients with stoma [22, 23]. The present study indicates that a low coloanal anastomosis might cause more sexual problems with negative impact on sexual functioning according to the EORTC QLQ-CR38 scores in the group without a stoma. On the other hand, in the stoma group, a parastomal hernia or bulge around the stoma significantly impaired sexual functioning and enjoyment. Male sexual functioning might be inferior in the group with a stoma, but the small group without stoma answering this question makes it difficult to interpret.

Good quality of life is important, but what it stands for is a very personal experience. Tools currently available to assess HRQoL describe what a group of patients experience, and conclusions about quality of life outcomes can be made on a group basis. However, each patient is an individual and HRQoL depends on factors such as social, emotional and religious background. Earlier studies have shown HRQoL to be reduced in stoma patients with further reduction if there is a bulge or a hernia around the stoma [23, 24]. A Cochrane report from 2012 challenges the opinion that stoma patients have an inferior HRQoL and calls for better prospective studies [9].

The patient’s own experience is important and knowledge of HRQoL can provide guidance when choosing between different treatments. The only treatment option to a very low rectal cancer might be APR to achieve radical surgery and cure, giving the patient a permanent stoma. This study supports the hypothesis that HRQoL is impaired by a stoma and it also reveals additional negative impact by a bulge or a hernia around the stoma. A stoma should be avoided when sphincter-preserving surgery is possible. A deeper and more careful knowledge about the possible impairment on HRQoL a stoma might cause will be helpful when informing and preparing the patient before surgery. This study also emphasizes the importance of finding an effective prevention and treatment of parastomal hernia.
